# Components of partial resistance to *Plasmopara viticola* enable complete phenotypic characterization of grapevine varieties

**DOI:** 10.1038/s41598-020-57482-0

**Published:** 2020-01-17

**Authors:** Federica Bove, Vittorio Rossi

**Affiliations:** Department of Sustainable Crop Production, Università Cattolica del Sacro Cuore, Via Emilia Parmense 84, 29122 Piacenza, Italy

**Keywords:** Plant sciences, Plant breeding, Plant genetics, Plant immunity, Plant stress responses

## Abstract

Six components of partial resistance (RCs) were studied in 15 grapevine varieties with partial resistance to *Plasmopara viticola*: (i) infection frequency (IFR, proportion of inoculation sites showing sporulation), (ii) latent period (LP50, degree-days between inoculation and appearance of 50% of the final number of sporulating lesions), (iii) lesion size (LS, area of single lesions in mm^2^), (iv) production of sporangia (SPOR, number of sporangia produced per lesion, and SPOR’, number of sporangia produced per mm^2^ of lesion), (v) infectious period (IP, number of sporulation events on a lesion), and (vi) infectivity of sporangia (INF, infection efficiency of sporangia produced on resistant varieties). Artificial inoculation monocycle experiments were conducted for a 3-year period on leaves collected at leaf development, flowering, and fruit development. Compared to the susceptible variety ‘Merlot’, the partially resistant varieties showed reduced IFR, longer LP, smaller LS, fewer SPOR and SPOR’, shorter IP, and lower INF. At leaf development, IFR, SPOR, and INF were higher and LP was shorter than at flowering and fruit development. RCs analysis through monocyclic experiments provides reliable assessments of the resistance response of grapevine accessions. The workload required for routine assessment in breeding programs could be reduced by measuring IFR and SPOR, while producing robust results.

## Introduction

Downy mildew (DM) of grapevine is a serious disease caused by the obligate, biotrophic Oomycete *Plasmopara viticola*^1^. *P. viticola* originated from North America, was introduced in Europe in the 1870 s^2^, and spread across the continent in the following years^1^.

The level of partial resistance to DM varies widely among *Vitis* species and cultivars^3,4^. The Eurasian grapevine *V. vinifera*, which is widely cultivated for its agronomic and quality traits, is generally susceptible to DM, whereas Asian and American *Vitis* species (e.g., *V. riparia* and *V. rupestris*) show varying degrees of resistance^5^ because of their coevolution with the pathogen. The partial resistance to *P. viticola* is conferred to grapevines by specific loci referred to as Rpvs (resistance to *P. viticola*)^6^; partial resistance has also been found in *V. vinifera* germplasm^7^.

Breeding programs have been implemented based on the hybridization of American *Vitis* spp. with *V. vinifera* for the introgression of resistance genes into the domesticated background of *V. vinifera*. Some of the breeding lines, after several cycles of backcrossing, gained more than 80% of *V. vinifera* genetic background^8^, and incorporated one or more (pyramided) Rpvs. Some reports, which characterized partially resistant varieties under vineyard conditions, indicate a reduction in the speed of DM epidemics on resistant varieties in comparison with susceptible grapevine ones^9,10^. Since the early 1900s, several partially resistant varieties with good grape quality and in some cases capable of producing a *vinifera*-like wine^11–13^ have been selected and released by breeders. The resistance response of grapevines to DM has been well investigated^7,10,14–21^. Resistance mechanisms include a hypersensitive response (HR), the synthesis and accumulation of polyphenols (i.e., stilbene and flavonoid phytoalexins), the production of reactive oxygen species, and callose deposition^14,22–24^.

Partial resistance may influence several stages of the infection cycle, including spore germination, penetration into the host tissue, colonization of the inner host tissue, the duration of latent and infectious periods, and sporulation^25^. These processes are called “components of partial resistance” and regulate the epidemic that can result from a chain of infection cycles during the host-growing season. In partial resistance, the phenotype is the result of different phases of the infection process in which resistance responses occur, and each phase is considered a component of resistance that contributes to the overall response^26^. The most studied components of resistance include reduced infection efficiency, a longer latent period, a shorter infectious period, reduced sporulation, and a smaller lesion size^27–30^. Resistance components have been analyzed^31^ for a number of pathosystems, including Cercospora leaf spot of sugar beet^29,32^, yellow rust (*Puccinia striiformis* f. sp. *hordei*) of barley^33^, rice blast (*Pyricularia oryzae*)^34^, rice sheath blight (*Rhizoctonia solani*)^35^, leaf rust (*Puccinia triticina*) of wheat^36^, *Fusarium* head blight of wheat^37^, and *Phoma* black stem of sunflower^38^. However, such analysis has not been conducted on the DM–grapevine pathosystem.

From an epidemiological point of view, components of partial resistance all help to reduce the apparent infection rate (*r*) of the epidemic, thus slowing disease progress^25,39–42^. The effects of partial resistance components (RCs) on the epidemic development have been investigated for groundnut rust^43^, potato late blight^44^, wheat stripe rust^45^, and sugar beet Cercospora leaf spot^32^.

The objective of this work was to measure the RCs to *P. viticola* in 15 *Vitis* accessions, most of them carry one or more Rpvs (www.vivc.de), or showing moderate susceptibility to downy mildew. For the sake of simplicity, these 15 accessions are referred to as “partially resistant varieties” in this paper. The following phenotypic traits were assessed in monocycle experiments conducted under environmentally controlled conditions: (i) degree of resistance, based on the OIV 452-1 descriptor^46^; (ii) infection frequency; (iii) duration of the latent period; (iv) lesion size; (v) number of sporangia produced per lesion and per lesion surface unit, (vi) duration of the infectious period; and (vii) infectivity of the sporangia produced on DM lesions.

## Results

### Degree of resistance in the tested varieties

**Figure 1:**
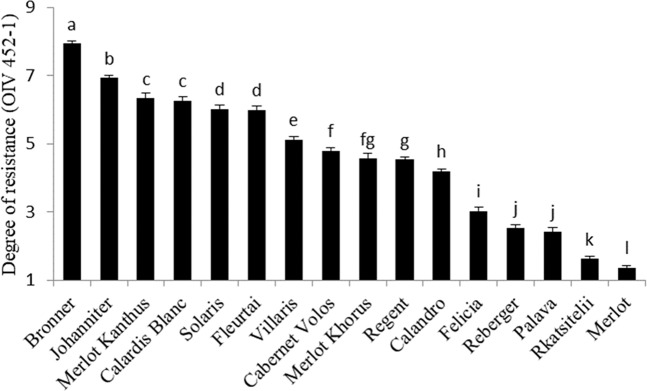
Resistance to *Plasmopara viticola* assessed on leaf discs of 16 grapevine varieties (X axis) after artificial inoculation. The degree of resistance is expressed according to the descriptor OIV 452-1^46^ : 1 = very low degree of resistance; 3 = low degree of resistance; 5 = medium degree of resistance; 7 = high degree of resistance; and 9 = very high degree of resistance. Values are means of data collected in a 3-year period (from 2014 to 2016) and at three growth stage, with 75 replicate leaf discs for each variety in each experiment + SE. Values with different letters are significantly different according to the LSD test (P =  0.05).

A factorial analysis of variance (ANOVA) for the degree of resistance to *P. viticola* according to the OIV 452-1 descriptor^46^ showed significant effects of year, growth stage, variety, and their interactions (P < 0.001). Year and growth stage accounted for 3.7% and 3.5% of total variance, respectively, while variety accounted for 63% (Supplementary Table S1). The susceptible control ‘Merlot’ scored 1.4 in resistance to DM (very low resistance). Among the tested varieties, the lowest OIV 452-1 value was 1.6 (for ‘Rkatsitelii’), and the highest values were 6.9 (for ‘Bronner’) and 7.9 (for ‘Johanniter’) (Fig. 1).

### Resistance components in ‘Merlot’ and in the partially resistant varieties

ANOVAs showed a significant effect (P < 0.001) of variety for all RCs (Table 1). Variety and its interactions accounted for 67% to 89% of the total variance, depending on the RC (Supplementary Table S1). Plant growth stage at the time of RC measurement also had a significant effect (P ≤ 0.01) on RCs; the interaction variety × growth stage was significant for some RCs but not for others, and accounted for only a small percentage of the total variance (Supplementary Table S1). Our analysis now focuses on the main effects of variety (Table 1) and growth stage (Table 2).

**Table 1:** Values of the components of partial resistance (RCs) to *Plasmopara viticola* measured for the grapevine varieties described in Table 5. Values are the average of the experiments carried out by inoculating grape leaf discs with a sporangial suspension of *P. viticola* in a 3-year period and at three growth stages of grapevines, with 75 replicate leaf discs per experiment. RCs are IFR = infection frequency (0–1); AUIPC = Area Under Infection Progress Curve; LP50 = latent period (degree days); LS = lesion size (mm^2^); SPOR’ = number of sporangia per mm^2^ lesion; SPOR = number of sporangia per downy mildew lesion; IP = infectious period (number of sporulation events); INF = infectivity of the sporangia produced on the variety and inoculated on ‘Merlot’ (0–1). P-values indicate the significance of the effect of the variety in the ANOVA, and CV% are the coefficients of variation. Means followed by the same letter in the same column are not significantly different according to the Fisher-protected LSD test (P = 0.05).

Variety	IFR		AUIPC		LP50		LS		SPOR’		SPOR		IP		INF	
‘Bronner’	0.54	k	3.2	i	111.0	b	3.0	fgh	20.2	jk	43	k	1.1	e	0.63	g
‘Cabernet Volos’	0.94	abc	5.9	bc	97.2	fg	6.6	def	75.9	h	450	fg	4.8	a	0.66	fg
‘Calandro’	0.95	ab	5.9	cd	101.7	d	1.4	h	665.4	c	930	d	2.7	bcd	0.94	a
‘Calardis Blanc’	0.76	i	4.7	g	99.2	ef	16.2	b	27.6	i	318	h	1.9	de	0.75	cdef
‘Felicia’	0.89	de	5.7	e	98.1	fg	5.6	fg	172.4	f	925	d	2.7	bcd	0.86	abc
‘Fleurtai’	0.85	ef	5.0	f	107.6	c	9.0	cd	19.9	k	99	j	4.8	a	0.80	bcde
‘Johanniter’	0.54	k	3.1	i	113.8	a	0.6	h	975.2	b	655	e	2.1	cd	0.69	efg
‘Merlot’	0.95	abc	6.2	b	94.8	h	21.1	a	344.7	de	7257	a	3.3	b	0.77	bcdef
‘Merlot Kanthus’	0.66	j	3.9	h	107.8	c	5.1	fg	16.0	k	45	k	1.9	de	0.71	efg
‘Merlot Khorus’	0.81	gh	5.1	f	101.6	de	9.7	c	26.4	ij	160	i	2.7	bcd	0.67	fg
‘Palava’	0.84	fg	5.1	f	103.0	d	9.5	cd	313.3	e	2840	c	2.2	cd	0.86	abc
‘Reberger’	0.89	d	5.5	e	103.0	d	2.8	gh	2158.7	a	5951	a	2.5	bcd	0.88	ab
‘Regent’	0.92	cd	5.7	de	99.3	ef	5.6	efg	108.4	g	550	ef	3.0	bc	0.83	bcd
‘Rkatsitelii’	0.97	a	6.5	a	90.5	i	11.0	c	419.5	d	4055	b	2.1	cd	0.72	defg
‘Solaris’	0.79	hi	4.9	fg	102.6	d	8.4	cde	89.0	gh	675	e	2.8	bcd	0.75	cdef
‘Villaris’	0.93	bc	5.9	cd	96.1	gh	5.7	efg	70.8	h	352	gh	4.9	a	0.85	abc
P-value	P < 0.001		P < 0.001		P < 0.001		P < 0.001		P < 0.001		P < 0.001		P < 0.001		P < 0.001	
CV %	16.8		19.5		6.0		70.6		161.3		142.3		39.4		11.8

**Table 2:** Values of the components of partial resistance (RCs) to *Plasmopara viticola* measured at three growth stages of grapevines, i.e. leaf development, flowering, and fruit development, corresponding to growth stages 18, 65, and 79, respectively^73^. Values are the average of the experiments carried out by inoculating grape leaf discs with a sporangial suspension of *P. viticola* in a 3–year period and 16 grapevine varieties, with 75 replicate leaf discs per experiment. RCs are IFR = infection frequency (0–1); AUIPC = Area Under Infection Progress Curve; LP50 = latent period (degree days); SPOR’ = number of sporangia per mm^2^ lesion; SPOR = number of sporangia per downy mildew lesion; INF = infectivity of the sporangia produced on the variety and inoculated on ‘Merlot’ (0–1). P-values indicate the significance of the effect of growth stage in the ANOVAs, and CV% values are the coefficients of variation. Means followed by the same letter in the same column are not significantly different according to Fisher-protected LSD test (P = 0.05).

Growth stage		IFR		AUIPC		LP50		SPOR’		SPOR		INF	
Leaf development	**18**	0.85	a	5.6	a	91.1	b	333	a	1768	a	0.88	a
Flowering	**65**	0.81	b	4.9	b	105.8	a	97	b	415	b	0.70	c
Fruit development	**79**	0.82	b	5.0	b	106.6	a	81	c	381	b	0.76	b
P-value		0.001		<0.001		<0.001		<0.001		<0.001		<0.001	
CV %		2.3		7.4		8.6		83.0		92.6		11.5	

The overall average values for the infection frequency (IFR) for ‘Rkatsitelii’, ‘Calandro’, ‘Cabernet Volos’, ‘Villaris’, and ‘Regent’ were not significantly different from that of the susceptible control ‘Merlot’, which had an average IFR value of 0.95 (Table 1). IFR values were lower for ‘Reberger’, ‘Felicia’, ‘Fleurtai’, ‘Palava’, ‘Merlot Khorus’, ‘Solaris’, ‘Calardis Blanc’, ‘Merlot Kanthus’, ‘Bronner’, and ‘Johanniter’ than for ‘Merlot’, with a reduction relative to ‘Merlot’ ranging from 6% for ‘Reberger’ to 43% for ‘Bronner’ and ‘Johanniter’ (Table 1). The average IFR value was significantly higher at growth stage 18, i.e. shoot growing (IFR = 0.85), than at stages 65, i.e. flowering (IFR = 0.81), or 79, i.e. fruit development (IFR = 0.82) (Table 2).

Area Under Infection Progress Curve (AUIPC) for ‘Cabernet Volos’ and ‘Rkatsitelii’ were significantly lower and higher, respectively, than the value for the susceptible control ‘Merlot’ (Table 1). AUIPC values for all of the other varieties were lower than the AUIPC for ‘Merlot’, with reductions relative to ‘Merlot’ ranging from 4% to 50%. AUIPC values were lowest for ‘Bronner’ and ‘Johanniter’. As was the case for IFR, the average AUIPC was significantly higher at stage 18 than at stages 65 or 79 (Table 2).

The duration of the latent period (LP50) did not significantly differ between ‘Villaris’ and the susceptible control ‘Merlot’ (Table 1). Relative to ‘Merlot’ and ‘Villaris’, the LP50 was shorter for ‘Rkatsitelii’ but was longer for all other varieties. The LP50 was longest for ‘Johanniter’ and ‘Bronner’, with an increase of 20% and 17%, respectively, relative to ‘Merlot’. The LP50 was significantly shorter at growth stage 18 than at the other two growth stages, which were not significantly different each other (Table 2).

The lesion size (LS) was smaller for all the partially resistant varieties than for ‘Merlot’ (Table 1), with reductions ranging from 23% to >90% (93% for ‘Calandro’ and 96% for ‘Johanniter’).

The number of sporangia produced per mm^2^ of DM lesion (SPOR’) was higher for ‘Reberger’, ‘Johanniter’, and ‘Calandro’ than for ‘Merlot’, and did not significantly differ among ‘Rkatsitelii’, ‘Palava’, and ‘Merlot’ (Table 1). For all other varieties, SPOR’ values were reduced by 50% to 95% compared to ‘Merlot’, and values were lowest values for ‘Bronner’, ‘Fleurtai’, and ‘Merlot Kanthus’. SPOR’ values were higher at stage 18 than at the other two stages (Table 2).

The number of sporangia produced per lesion (SPOR) on ‘Reberger’ was not significantly different from the number on ‘Merlot’ (Table 1). In the partially resistant varieties, the SPOR was reduced by 44% to 99% compared to ‘Merlot’. SPOR was lowest on ‘Bronner’, ‘Merlot Kanthus’, and ‘Fleurtai’. Like SPOR’ values, SPOR values were higher at stage 18 than at the other two stages (Table 2).

The infectious period (IP) for ‘Regent’, ‘Solaris’, ‘Felicia’, ‘Calandro’, ‘Merlot Khorus’, and ‘Reberger’ was not significantly different from that for ‘Merlot’ (Table 1), while the IP was longer for ‘Villaris’, ‘Cabernet Volos’, and ‘Fleurtai’. For ‘Palava’, ‘Johanniter’, ‘Rkatsitelii’, ‘Merlot Kanthus’, ‘Calardis Blanc’, and ‘Bronner’, the number of sporulation events was 1/3 to 2/3 lower than for the control ‘Merlot’.

For most varieties, the infectivity of sporangia produced on the DM lesions (INF) did not significantly differ from that of the sporangia produced on ‘Merlot’ (Table 1). INF, however, was significantly higher for ‘Calandro’ (by 22%) and significantly lower for ‘Bronner’ (by 19%). INF was lower at stage 65 than at the other two stages (Table 2).

Overall, the variability (as indicated by the coefficient of variability, CV) among grapevine varieties was high for SPOR’, LS, and SPOR but was low for LP50 (Table 1).

### Relationships among resistance components

As indicated by the correlation matrix (Table 3), there was a significant (P < 0.05) relationship between IFR (and AUIPC as a consequence), LP50, IP, and INF, indicating that as IFR decreased, LP50 increased and IP and INF decreased. LS was significantly (P = 0.032) correlated with LP50, so that LS at 11 dpi increased as the time for the lesions to appear shortened. SPOR’, in contrast, was not correlated with any of the other RCs, with the exception for SPOR.

**Table 3:** Correlation coefficients (r values) among the components of partial resistance (RCs) to *Plasmopara viticola* measured for the grapevine varieties described in Table 5. RCs are: IFR = infection frequency (0–1); AUIPC = Area Under Infection Progress Curve; LP50 = latent period (degree days); LS = lesion size (mm^2^); SPOR’ = number of sporangia per mm^2^ lesion; SPOR = number of sporangia per downy mildew lesion; IP = infectious period (number of sporulation events); INF = infectivity of the sporangia produced on the variety and inoculated on ‘Merlot’ (0–1). RCs were measured in monocycle experiments conducted under environment-controlled conditions, by inoculating a sporangial suspension of a *P. viticola* population on leaf discs. The P-value is shown for each correlation coefficient. Values corresponding to significant correlations (P < 0.01 and P < 0.05) are in bold.

		IFR	AUIPC	LP50	LS	SPOR’	SPOR	IP	INF
IFR	r	1	**0.989**	**−0.846**	0.325	0.059	0.414	**0.569**	**0.564**
	P-value		<0.001	<0.001	0.22	0.828	0.111	0.021	0.023
AUIPC	r		1	**−0.905**	0.372	0.044	0.456	**0.508**	**0.498**
	P-value			<0.001	0.156	0.871	0.076	0.045	0.05
LP50	r			1	**−0.538**	0.132	−0.407	−0.327	−0.244
	P-value				0.032	0.627	0.117	0.216	0.363
LS	r				1	−0.338	0.433	0.1	−0.127
	P-value					0.2	0.094	0.713	0.639
SPOR’	r					1	**0.579**	−0.181	0.363
	P-value						0.019	0.502	0.167
SPOR	r						1	−0.078	0.258
	P-value							0.775	0.335
IP	r							1	0.209
	P-value								0.438
INF	r								1
	P-value								

### Cluster analysis

**Figure 2:**
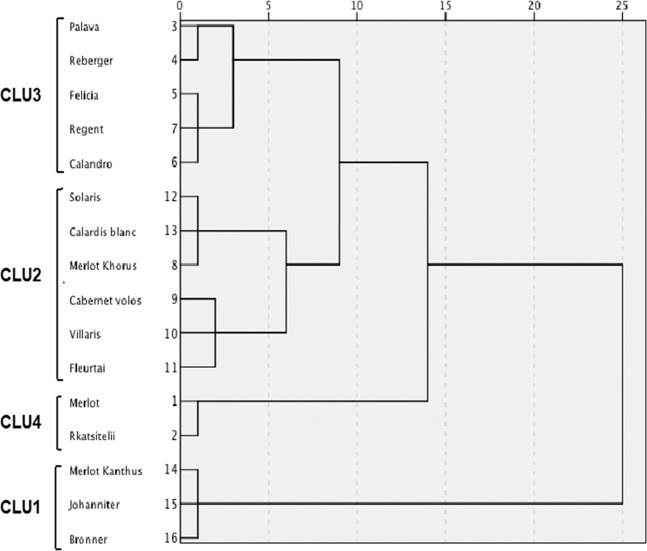
Dendrogram resulting from a hierarchical cluster analysis of the five components of partial resistance (RCs) to *Plasmopara viticola* measured in the grapevine varieties described in Table 5. The RCs are infection frequency (IFR), latency period (LP50), sporulation (SPOR), infectious period (IP), and the infectivity of the sporangia produced on the variety and inoculated on Merlot (INF). Four clusters were identified in the dendrogram (CLU1 to CLU4).

The hierarchical cluster analysis grouped the varieties at different rescaled distances (Fig. 2); at the intermediate distance, four clusters were identified. Based on the OIV degree of resistance (standard OIV 452-1), these clusters represent “high resistance” (CLU1, with an average degree of resistance OIV = 7.0), “medium resistance” (CLU2, OIV = 5.4), “low resistance” (CLU3, OIV = 3.3), and “very low resistance” (CLU4, OIV = 1.5); no varieties showed a “very high” degree of resistance (i.e., with OIV = 9). The average values for each RC of each cluster are shown in Table 4. The among-cluster ANOVA revealed significant differences (P < 0.002) for IFR, LP50, SPOR, and INF, but not for IP (P = 0.07). CLU1 included ‘Bronner’, ‘Johanniter’, and ‘Merlot Kanthus’; CLU1 had the lowest IFR value, the longest LP50, the lowest SPOR, the shortest IP, and the smallest INF. In contrast, CLU4, which included the susceptible control ‘Merlot’ and ‘Rkatsitelii’, had the highest IFR, the shortest LP50, and the highest SPOR. CLU3 contained five varieties (‘Palava’, ‘Reberger’, ‘Felicia’, ‘Calandro’, and ‘Regent’) and had higher IFR, SPOR, and INF values than CLU2, which contained six varieties (‘Merlot Khorus’, ‘Cabernet Volos’, ‘Villaris’, ‘Fleurtai’, ‘Solaris’, and ‘Calardis Blanc’).

**Table 4:** Values of five components of partial resistance (RCs) to *Plasmopara viticola* for the four clusters in Fig. 2; OIV ratings and corresponding degrees of resistance are also indicated. Values are the average of the experiments carried out by inoculating grape leaf discs with a sporangial suspension of *P. viticola* in a 3-year period and 3 growth stages of grapevines, with 75 replicate leaf discs per experiment. RCs are: IFR = infection frequency (0–1); LP50 = latent period (degree days); SPOR = number of sporangia per downy mildew lesion; IP = infectious period (number of sporulation events); INF = infectivity of the sporangia produced on the variety and inoculated on ‘Merlot’ (0–1). OIV is the OIV descriptor 452–1 for the degree of resistance, which ranges from 1 to 9, corresponding to very low to very high resistance, respectively. P-values indicate the significance of the effect of cluster in the ANOVAs. Means followed by the same letter in the same column are not significantly different according to Fisher-protected LSD test (P = 0.05).

Cluster	RCs										OIV		Resistance
	IFR		LP50		SPOR		IP		INF				
CLU4	0.96	a	92.7	c	6604	a	2.7	—	0.75	b	1.5	a	Very low
CLU3	0.90	ab	101.0	b	1684	b	2.6	—	0.87	a	3.3	b	Low
CLU2	0.84	b	100.7	b	477	b	3.7	—	0.75	b	5.4	c	Medium
CLU1	0.58	c	110.9	a	272	b	1.7	—	0.67	b	7.0	d	High
P-value	<0.001		0.001		<0.001		0.07		0.002		<0.001		

**Figure 3:**
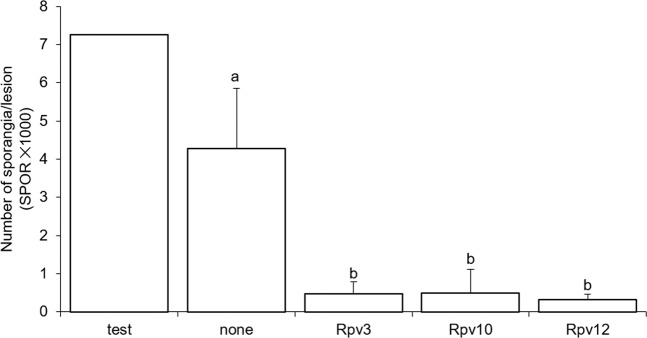
Number of sporangia per downy mildew lesion (SPOR) measured on the grapevine varieties described in Table 5. Varieties were assigned to the following five groups: susceptible control ‘Merlot’ (test), resistant varieties with no Rpv (none), or with Rpv3, Rpv10 or Rpv12. Values are means of data collected in a 3-year period and at three growth stage of grapevines, with 75 replicate leaf discs for each variety in each experiment + SE. Values with different letters are significantly different according to the LSD test (P  = 0.05).

CLU4 contained two varieties without Rpvs, while CLU3 contained varieties without Rpvs or with Rpv3. Rpv3 was also present, alone or pyramided with other Rpvs, in two varieties of CLU2 and two of CLU1. Similarly, Rpv10 was present in two varieties belonging to CLU1 and to CLU2, respectively. The three varieties with Rpv12 were all in CLU2. According to ANOVAs, the RCs IFR, LP50, IP, and INF were not significantly affected by the presence of a particular Rpv in the genome (P > 0.05, Supplementary Table S2). On the contrary, SPOR was significantly higher in the susceptible control ‘Merlot’ than in the varieties with no Rpvs and in the partially resistant varieties carrying Rpv3, Rpv10, or Rpv12; SPOR did not significantly differ among partially resistant varieties carrying Rpv3, Rpv10, or Rpv12 (Fig. 3).

## Discussion

The purpose of the present research was to characterize the partial resistance to downy mildew (DM) in 15 grapevine varieties, most of which carried one Rpv from *V. rupestris* or *V. amurensis*^14,47–49^. Resistance was assessed relative to a susceptible control, ‘Merlot’. The study was conducted by inoculating leaf discs with *P. viticola*, which is a widely used bioassay in the study of genetic resistance to DM in grapevines^7,9,19,50–52^. Inoculations were performed by using a suspension of *P. viticola* sporangia derived from sporangia collected in multiple vineyards and then maintained on the susceptible variety ‘Merlot’ for the duration of the study. A population of *P. viticola* was used instead of single isolates in order to minimize the effect of host–strain genotype interaction on the phenotypic evaluation of the resistance components^53–55^.

The study assessed six main resistance components (RCs) that cover the entire disease monocycle and that do not overlap in terms of processes^26,31^. RCs were expressed according to their proper dimensions (e.g., frequency for IFR, thermal time for LP50, or numbers of sporangia for SPOR).

IFR was measured as the proportion of inoculated sites that produced DM lesions in the leaf discs assay. IFR is a proxy of infection efficiency (IE), which has been measured as the number of DM lesions generated by each zoospore deposited on the leaf surface^56^. IE was not used in the current study because its measurement is too complex^56^ given the number of experiments that were performed. In addition, IE is biased because the number of zoospores produced per sporangium is variable^56^, multiple germ tubes (from multiple zoospores) can penetrate the same stoma^57^, and a single lesion may result from multiple penetrations^23^. Reduction of infection efficiency (measured as IFR in this work) reflects the ability of the plant to reduce the number of pathogen propagules that successfully go through the different steps of the infection process, e.g., the ability of the plant to prevent germ tube penetration of stomata, the formation of substomatal vesicles, the growth of primary and secondary hyphae in the mesophyll, or the growth and functioning of haustoria. IFR can involve both pre- and post-infection mechanisms, such as callose deposition in stomata^58–60^, the presence of inner cuticular rims^22^, and a HR^14,60,61^.

The duration of the latent period indicates the time required for the pathogen to produce sporangia on the lesion surface. A longer latent period may result from reduced hyphal growth in the mesophyll of the host plant due to the thickness of the spongy mesophyll and cell walls^4,22,62^. A longer latent period may also result from a reduced number or functioning of haustoria resulting from HR^14^, such that the flow of nutrients from host cells to the intercellular mycelium is reduced. AUIPC was also calculated to account for the observation that DM lesions appeared later in partially resistant varieties than in the susceptible control. AUIPC is therefore related to the duration of the incubation period, which is the time required for lesions to appear. The incubation period is not a resistance component *sensu* Zadoks^31^ or Parlevliet^40^, because it does not affect the epidemic development and it is part of the latent period.

The other RCs (i.e., the number of the sporangia produced per DM lesion and per area of DM lesion, the duration of the infectious period, and the infectivity of the sporangia produced on DM lesions) may also be related to a reduced flow of nutrients from the host to the pathogen. For instance, reduced sporangiophore density and reduced numbers of sporangia produced per unit of leaf area were observed in ‘Bianca’ as a consequence of an HR^14^. The size of DM lesions also reflects the ability of the host to limit the growth of the pathogen in the mesophyll. The synthesis of resveratrol and flavonoids, the increased peroxidase activity, and the formation of lignin in the host tissue all restrict the development of *P. viticola* and result in smaller lesions^63^.

INF was not previously considered in analyses of resistance components^31^, but reduced INF may slow the progress of DM epidemics in partially resistant varieties. The infectivity of *P. viticola* sporangia produced on DM lesions on leaves of partially resistant varieties has been poorly studied. However, finding similar to those for DM in the current study were described for leaf rust (*Puccinia triticinia*) on wheat^64^. Delmotte *et al*.^55^ observed that cross-inoculation with the sporangia produced on the partially resistant variety ‘Regent’ on the susceptible cultivar ‘Cabernet Sauvignon’ resulted in higher disease severity, higher sporangia production, and smaller sporangia than inoculation with sporangia produced on the susceptible variety. This seems in contrast with the findings of the current study, in which the sporangia produced on ‘Regent’ had lower infectivity on the susceptible ‘Merlot’ than the sporangia produced on ‘Merlot’. These contrasting results might be explained by differences in methods or in the genotypes of both the host plant and the pathogen.

RCs were expressed to different degrees among the 15 varieties, with significant differences relative to the susceptible ‘Merlot’. High among-variety variability was found for SPOR’, LS, and SPOR (i.e., the combination of LS and SPOR’), and low among-variety variability was found for IP. As expected, lower infection frequency, longer latent period, reduced sporulation, shorter infectious period, and lower infectivity of sporangia were found in the partially resistant varieties than in the susceptible ‘Merlot’. Changes in some of these RCs were significantly correlated, so that varieties with low IFR values also had long LP50 values and low IP and INF values (Table 3). Similar results were found for yellow rust (*Puccinia striiformis* f. sp. *hordei*) on barley^33^, leaf rust (*Puccinia triticina*) on wheat^36^, and *Phoma* black stem of sunflower^38^, indicating that the mechanisms of resistance act on different stages of the infection process, as previously discussed. Surprisingly, sporulation was not correlated with the other RCs, and this requires further investigation.

Expression of resistance components was generally highest in the varieties carrying Rpv3, Rpv10 or Rpv12, which all grouped in the clusters of high and medium resistance (based on the OIV standards), i.e. CLU1 and CLU2, respectively; the exceptions were ‘Felicia’, ‘Calandro’, and ‘Regent’, which were in the cluster with low resistance (i.e. CLU3), even though they carry Rpv3 (Fig. 2; Table 4). Unfortunately, no association was found between RCs and the Rpvs considered in this work, which would been helpful in breeding programs. On the one hand, the highest level of resistance was found in ‘Bronner’, ‘Merlot Kanthus’ and ‘Johanniter’ (belonging to the same CLU1). ‘Bronner’ (carrying Rpv10) and ‘Johanniter’ (Rpv3) are known to be resistant to DM^10,65,66^, and both show an HR to *P. viticola* that leads to cell necrosis and reduced production of sporangia^14,67^. ‘Merlot Kanthus’ also carries Rpv3. On the other hand, ‘Calandro’, ‘Regent’, and ‘Felicia’ also carry Rpv3, but the resistance of these varieties was low in this work, as already observed in other studies^3,10,65^. The resistance expressed by ‘Solaris’ (CLU2, medium resistance level) involves both callose synthesis and an HR^60^. The varieties ‘Merlot Khorus’, ‘Cabernet Volos’, and ‘Fleurtai’ carry the Rpv12 (grouped in CLU2), inherited from *V. amurensis*^49^. A typical resistance response of *V. amurensis* consists in the activation of physical mechanisms involving callose deposition^15,58^ that leads to the degeneration of portions of mycelium and the alteration of sporangiophore shape^7^. ‘Rkatsitelii’, a Georgian grapevine variety, showed a very low level of resistance to DM, confirming the results of Bitsadze *et al*.^68^.

Overall, our findings indicate that the routine measurement of RCs in breeding programs can be simplified by the measurement of only IFR and SPOR. Table 4 shows that the assessment of IFR made it possible to distinguish CLU2 from CLU1 (medium and high resistance level, respectively), and from CLU4 (very low resistance level), but not CLU3 (low resistance level) from CLU4. At the same time, SPOR made it possible to distinguish CLU4 from CLU3. Therefore, IFR and SPOR, together, were able to distinguish all the four clusters. In addition, since IFR and SPOR are the major drivers of DM epidemic progress in vineyards^69^, which is caused by the concatenation of infection cycles, the phenotyping of partial resistant varieties by using these two RCs in monocyclic experiments would also account for their effect on epidemic development. This would reduce the workload of phenotyping while still producing correct assessment of resistance.

The current research documented differences in resistance expression among growth stages, with lowest resistance at stage 18, i.e., at leaf development. To our knowledge, no previous studies have reported this growth stage-related variability, and such variability requires further investigation. It is well known from Van der Plank’s^25^ studies of resistance to potato late blight (*Phytophthora infestans*) that resistance responses are inducible, which suggests that the expression of resistance in response to attack might be costly^70^. The metabolic cost of defence may be a form of allocation cost, in which resources devoted to self-protection are not available for other activities such as growth or reproduction^71^. Because fungicides must be used to protect even partially resistant grapevine varieties against DM^51,72^, a better understanding of the temporal dynamics of resistance expression may be useful in scheduling these fungicide applications.

## Materials and Methods

### Plant material

Fifteen partially resistant grapevine varieties and the *V. vinifera* variety ‘Merlot’, which is highly susceptible to downy mildew and served as a positive control, were used. The varieties, their pedigree, and their Rpvs are listed in Table 5. ‘Calardis Blanc’, ‘Felicia’, ‘Villaris’, ‘Calandro’, ‘Regent’, and ‘Reberger’ were developed by the Julius Kühn Institut (JKI) in Geilweilerhof, Siebeldingen (Germany). ‘Bronner’, ‘Johanniter’, and ‘Solaris’ were developed at the Institute of Viticulture and Enology in Freiburg (Germany). ‘Merlot Khorus’, ‘Merlot Kanthus’, ‘Cabernet Volos’, and ‘Fleurtai’ were developed at the University of Udine and Institute of Applied Genetics (IGA) in Italy.

**Table 5:** The 16 varieties used in this study, their pedigrees, and their resistance-related loci (Rpv).

Variety	Pedigree	Loci				
		Rpv3	Rpv3^1^	Rpv3^2^	Rpv10	Rpv12
‘Bronner’	Merzling × Geisenheim 6494	—	—	—	x	—
‘Cabernet Volos’	Cabernet sauvignon × 20/3	—	—	—	—	x
‘Calandro	Domina × Regent	—	x	—	—	—
‘Calardis blanc’	Geilweilerhof GA-47-42 × S.V. 39-639 KL.1	—	x	x	—	—
‘Felicia’	Sirius × Vidal Blanc	x	—	—	—	—
‘Fleurtai’	Tocai × 20/3	—	—	—	—	x
‘Johanniter’	Riesling Weiss × Freiburg 589-54	—	x	—	—	—
‘Merlot’		—	—	—	—	—
‘Merlot Kanthus’	Merlot × 20/3	x	—	—	—	—
‘Merlot Khorus’	Merlot × 20/3	—	—	—	—	x
‘Palava’	Traminer × Mueller Thurgau	—	—	—	—	—
‘Reberger’	Regent × Lemberger	—	—	—	—	—
‘Regent’	Diana × Chambourchin	—	x	—	—	—
‘Rkatsiteli’	Unknown	—	—	—	—	—
‘Solaris’	Merzling × Geisenheim 6493	—	—	—	x	—
‘Villaris’	Sirius × Vidal blanc	—	x	—	—	—

These partially resistant varieties and the positive control ‘Merlot’ were grown in three experimental vineyards in Northern Italy; one vineyard was in Ferrara di Monte Baldo (45°41′5.23′′N 10°51′52.81′′E), and two were in Piacenza (PC, 35°01′32.76′′N 9°39′09.82′′E and 45°02′05.83′′N 9°43′46.41′′E). In 2014, these vineyards were 3, 3, and 2 years old, respectively. Each variety was managed with a single Guyot training system and without fungicide treatment for the duration of the experiment. The experiment was conducted for a 3-year period, from 2014 to 2016.

In each vineyard and year, five plants of each variety were randomly selected at three growth stages: (i) shoot growing, (ii) flowering, and (iii) fruit development. These corresponded to the growth stages 18, 65, and 79, respectively, of Lorenz *et al*.^73^. At each growth stage, the fourth leaf from the apex of an actively growing shoot of each plant was sampled^74^. The detached leaves were then pooled, yielding 15 leaves per variety per stage.

The leaves were placed in a cooler (about 5 °C) and immediately transported to the laboratory where they were washed under tap water, disinfested with sodium hypochlorite (1%) for 1 minute, triple-rinsed using sterile demineralized water, and finally arranged under a sterile laminar flow until their surfaces were completely dry. Following Staudt & Kassemeyer^75^ and Rumbolz *et al*.^76^, 5 leaf discs, 21 mm in diameter, were cut from each leaf using a cork borer, so that 75 leaf discs were obtained for each variety (15 leaves, 5 leaf discs per leaf). The leaf discs were placed abaxial side up in Petri dishes (9 cm diameter) containing two layers of filter paper moistened with 3 ml of sterile demineralized water; the leaf discs were placed on nylon mesh to prevent direct contact with the moistened paper.

### Inoculum preparation

For the preparation of the inoculum to be used for artificial inoculations, field-produced sporangia were randomly collected and pooled with the aim of obtaining a diverse natural population of *P. viticola* sporangia and thereby minimizing the effect of a possible host × strain interaction on the phenotypic evaluation of the resistance components^54^. To obtain these sporangia, field-grown grape leaves showing typical DM lesions with fresh and abundant sporulation were collected in the spring of 2014 from several vineyards that had not been sprayed with fungicides; these leaves were collected from different *V. vinifera* varieties and from various locations in Northern Italy. The pooled leaves were brought to the laboratory, where sporangia were collected from lesions (with the help of a needle) and suspended in sterile double-distilled water. Droplets (10 μl) of this bulk suspension of sporangia were placed on the abaxial side of young, fresh leaves that had been detached from potted plants of ‘Merlot’ grown under isolation in a greenhouse at the University of Piacenza. The inoculated leaves were incubated for 7 days in a growth chamber at 20 °C and with high humidity and a 12-h photoperiod. The fresh sporangia produced on the DM lesions were suspended in sterile water, and the suspension was adjusted to 5 × 10^5^ sporangia ml^−1^. Immediately after preparation, these sporangial suspensions were used for the artificial inoculations described in the next section. The same suspensions were used to maintain the inoculum through repeated inoculations of Merlot leaves, using the same previously described method.

### Inoculations

For inoculation of the leaf discs in Petri dishes, four 10-µl droplets of sporangial suspension were applied to each leaf disc using a micropipette. Petri dishes containing the inoculated leaf discs were then sealed with Parafilm (to maintain a saturated atmosphere) and incubated for 24 h in a growth chamber at 20 °C and with a 12-h photoperiod. The inoculum drops were then dried using blotting paper but without touching the leaf surface^46^. Petri dishes were then sealed again and kept in the growth chamber until the components of partial resistance were measured.

### Assessment of resistance

The degree of resistance was visually assessed 11 days post inoculation (dpi) using a modified OIV descriptor 452-1^46^. Leaf discs were rated from the most susceptible to the most resistant as follows: 1, dense sporulation at all four inoculation sites (i.e., very low degree of resistance); 3, dense sporulation at two or three inoculation sites (low resistance); 5, sparse sporulation at two or three inoculation sites (medium resistance); 7, sparse sporulation at one inoculation site (high resistance); and 9, absence of sporulation (very high resistance).

### Measurement of the components of partial resistance

Leaf discs were examined daily with a stereomicroscope at 10-fold magnification for the assessment of the RCs as described in the following paragraphs. The 10-fold magnification allowed the screening of a high number of leaf discs with a resolution adequate to observe single sporangiophores of *P. viticola*^50^. No new DM signs appeared before 4 dpi or after 11 dpi.

#### Infection frequency

IFR was assessed at 11 dpi as the proportion of inoculation sites (i.e., sites where the inoculum drops were placed) showing typical DM lesions with sporulation^77^.

To account for the delay in lesion appearance following inoculation, the AUIPC was calculated by using the daily assessments of IFR between 4 and 11 dpi as follows:

1 $${\rm{AUIPC}}=\mathop{\sum }\limits_{i=1}^{{N}_{i}-1}\,\frac{({y}_{i}+\,{y}_{i+1})}{2}({t}_{i+1}-{t}_{i})$$

where: $({y}_{i}+\,{y}_{i+1})$ represents the sum of two consecutive values of IFR, and $({t}_{i+1}-\,{t}_{i})$ is the time interval between two consecutive assessments. The calculation follows the trapezoidal method for the estimation of the AUDPC (Area Under the Disease Progress Curve), which enables the discretization of the time variable and the calculation of the average disease intensity between each pair of adjacent time points^78^.

#### Duration of the latent period

The duration of the latent period (i.e., the time elapsed between inoculation and the start of sporulation on DM lesions) was measured as the thermal time^79^, i.e., as degree-days (DDs, base 0 °C) accumulated between the inoculation time and the time when 50% of the inoculation sites resulted in DM lesions at the end of the observation period (i.e., at 11 dpi); this is termed the LP50^80^. For instance, if IFR = 0.25 at 6 dpi (DD = 120, i.e., 6 days at 20 °C) and IFR = 0.75 at 7 dpi (DD = 140) on a leaf disc, then IFR = 0.5 is assumed to be at 6.5 dpi, corresponding to 130 DD; therefore, LP50 = 130.

#### Lesion size

At 11 dpi, a random sample of 50 leaf discs per variety, which have been inoculated in different growth stages and years, was photographed (13 megapixel f/1.9 resolution), and the area (in mm^2^) of each lesion (referred as LS) was determined using Assess 2.0 (Image analysis software for plant disease quantification, by Lakhdar Lamari, APS PRESS, Saint Paul, Minnesota). Example of calculation of the lesion size of downy mildew lesions on leaf discs is provided in Supplementary Fig. S1.

#### Production of sporangia

Production of sporangia was determined as the number of sporangia produced per DM lesion (SPOR) and per mm^2^ of DM lesion (SPOR’) at 11 dpi. Sporangia were carefully collected from each leaf disc by using a needle; the collected sporangia were suspended in 100 µl of sterile-demineralized water; the suspension was shaken for 10 s, and the sporangia were counted using a hemocytometer. The total number of sporangia was then divided by the number of DM lesions assessed at 11 dpi on each leaf disc (for SPOR), and by the area of each lesion (for SPOR’).

#### Duration of the infectious period

After 11 dpi, sporulating structures (sporangiophores and sporangia) were gently removed from leaf discs, which have been inoculated at fruit development in the second year, using a sterile cotton swab. Leaf discs were then incubated as previously described and observed every 2 days with a stereomicroscope (10 × magnification). When sporangiophores bearing sporangia re-appeared on lesions, sporulating structures were removed as previously described and the leaf discs were incubated again. This procedure was repeated until the lesions stopped producing new sporangia. The duration of the infectious period (IP, i.e., the period during which a lesion is fertile and continues to produce sporangia) was then expressed as the number of times the lesions produced sporangia^81,82^.

#### Infectivity of the sporangia produced on DM lesions

INF was assessed as the ability of the sporangia produced on DM lesions to cause infection on leaves of the susceptible variety ‘Merlot’. At 11 dpi, sporangia produced on the DM lesions obtained in the second year were suspended in water as previously described for inoculum preparation, and the suspension was adjusted to 10^3^ sporangia/ml. These sporangial suspensions were used to inoculate 15 leaf discs excised from ‘Merlot’ leaves that were collected and maintained as previously described. At 11 dpi, INF was assessed as the proportion of inoculation sites (i.e., sites where the inoculum drops have been placed) showing typical DM lesions with sporulation.

### Data analysis

The ANOVA was performed to determine whether RCs were significantly affected by grapevine variety, growth stage of vines, year (all considered as fixed factors), and their interactions. Before ANOVAs were conducted, SPOR and SPOR’ data were transformed using the natural logarithm to stabilize variances and were then back-transformed using the inverse exponential function. When F values were significant, means were compared using the Fisher’s protected least significant difference (LSD) *post hoc* test at P = 0.05.

To study the relationships among RCs, Pearson correlation coefficients were calculated between IFR versus AUIPC, LP50, LS, SPOR’, SPOR, IP, and INF.

To study the relationships between RCs and Rpvs, and among Rpvs, an ANOVA was performed by assigning the varieties into the following groups: susceptible control; varieties with no Rpvs; with Rpv3 (whether Rpv3^1^ and Rpv3^2^); with Rpv10; or with Rpv12.

A hierarchical cluster analysis was conducted for grouping grapevine varieties based on the within-group similarities and between-group differences in the average values of five RCs: IFR, LP50, SPOR, IP, and INF. AUIPC was not included in this analysis because it was closely correlated to IFR (see Table 3); LS and SPOR’ were also not included because SPOR incorporates the information concerning the lesion size and the sporulation per surface unit of lesions. The Ward’s method was used for clustering, in which the distance between two clusters is indicated by the increase in the sum of squares caused by merging of the clusters, with the Euclidean square distance for measuring similarities. Because the RC values were measured on different scales, some being much larger than others, the data were standardized by using the z-scores as follows: $({{\rm{x}}}_{{\rm{i}}}-\bar{{\rm{x}}})/{\rm{sd}}$, where ${{\rm{x}}}_{{\rm{i}}}$ is any value of a variable, $\bar{{\rm{x}}}$ is the average for the variable, and sd is the standard deviation. An ANOVA was then performed to determine whether the groups of varieties formed by the cluster analysis were significantly different for each of the five RCs.

All analyses were performed using IBM SPSS software version 24 (SPSS, Chicago, IL).

## Supplementary information

Supplementary information.

## Data Availability

The datasets generated during and/or analysed during the current study are available from the corresponding author on reasonable request.
