# Supplementary material for: Components of partial resistance to Plasmopara viticola enable complete phenotypic characterization of grapevine varieties
Source: Sci Rep. 2020 Jan 17;10:585. doi: 10.1038/s41598-020-57482-0 (PMC6969139; doi:10.1038/s41598-020-57482-0)

Components of partial resistance to *Plasmopara viticola* enable complete phenotypic characterization of grapevine varieties

Federica Bove[1], Vittorio Rossi[1]*

[1]Department of Sustainable Crop Production, Università Cattolica del Sacro Cuore, Via Emilia Parmense 84, 29122 Piacenza, Italy.

Correspondence and requests for material should be addressed to Vittorio Rossi (email: vittorio.rossi@unicatt.it; Tel. +39 0523 599253)

ORCID authors: F. Bove: 0000-0001-7635-7696; V. Rossi: 0000-0003-4090-6117

Abstract

Six components of partial resistance (RCs) were studied in 15 grapevine varieties with partial resistance to *Plasmopara viticola*: i) infection frequency (IFR, proportion of inoculation sites showing sporulation), ii) latent period (LP50, degree-days between inoculation and appearance of 50% of the final number of sporulating lesions), iii) lesion size (LS, area of single lesions in mm^2), iv) production of sporangia (SPOR, number of sporangia produced per lesion, and SPOR', number of sporangia produced per mm^2 of lesion), v) infectious period (IP, number of sporulation events on a lesion), and vi) infectivity of sporangia (INF, infection efficiency of sporangia produced on resistant varieties).

Artificial inoculation monocycle experiments were conducted for a 3-year period on leaves collected at leaf development, flowering, and fruit development.

Compared to the susceptible variety 'Merlot', the partially resistant varieties showed reduced IFR, longer LP, smaller LS, fewer SPOR and SPOR', shorter IP, and lower INF. At leaf development, IFR, SPOR, and INF were higher and LP was shorter than at flowering and fruit development.

RCs analysis through monocyclic experiments provides reliable assessments of the resistance response of grapevine accessions. The workload required for routine assessment in breeding programs could be reduced by measuring IFR and SPOR, while producing robust results.

Key Words: components analysis, downy mildew, grapevine, plant-pathogen interactions, *Plasmopara viticola,* resistance components, Rpv.

Supplementary materials

Table S1: Results of the analysis of variance for the OIV descriptor and for components of partial resistance (RCs) to *Plasmopara viticola* measured for the 16 grapevine varieties described in Table 5. Values are the average of the experiments carried out by inoculating grape leaf discs with a sporangial suspension of *P. viticola* in a 3-year period and at three growth stages of grapevines, with 75 replicate leaf discs per experiment. RCs are IFR = infection frequency of the sporangia produced on the susceptible control Merlot; AUIPC = Area Under Infection Progress Curve; LP50 = latent period; LS = lesion size; SPOR' = number of sporangia per mm^2 lesion; SPOR = number of sporangia per downy mildew lesion; IP = infectious period; INF = infectivity of the sporangia produced on the variety and inoculated on Merlot. P-values indicate the significance level and % the percentage of total variance accounted by main factors (year, growth stage and variety) and their interactions.

	**OIV**		**IFR**		**AUIPC**		**LP50**		**LS**		**SPOR'**		**SPOR**		**IP**		**INF**	
	P-value	%	P-value	%	P-value	%	P-value	%	P-value	%	P-value	%	P-value	%	P-value	%	P-value	%
**Year**	<0.001	3.68	<0.001	0.51	0.014	0.12	<0.001	2.35	-	-	0.111	0.04	0.063	0.07	-	-	-	-
**Growth stage**	<0.001	3.55	0.001	0.33	<0.001	3.27	<0.001	26.43	-	-	<0.001	10.42	<0.001	11.68	-	-	<0.001	11.31
**Variety**	<0.001	62.67	<0.001	48.07	<0.001	47.63	<0.001	22.42	<0.001	-	<0.001	59.80	<0.001	51.14	<0.001	-	<0.001	25.02
**Year × Growth stage**	<0.001	0.39	<0.001	1.16	<0.001	0.38	<0.001	3.98	-	-	<0.001	0.62	0.002	0.25	-	-	-	-
**Year × Variety**	<0.001	6.27	<0.001	4.95	<0.001	4.82	<0.001	8.50	-	-	<0.001	6.48	<0.001	8.51	-	-	-	-
**Growth stage × Variety**	<0.001	15.38	<0.001	23.92	<0.001	24.24	<0.001	23.51	-	-	<0.001	15.85	<0.001	21.04	-	-	<0.001	63.67
**Year × Growth stage × Variety**	<0.001	8.07	<0.001	21.05	<0.001	19.54	<0.001	12.82	-	-	<0.001	6.78	<0.001	7.31	-	-	-	-

Table S2: Infection frequency (IFR, 0-1), latent period (LP50, degree days), number of sporangia per downy mildew lesion (SPOR), infectious period (IP, number of sporulation events), and infectivity of the sporangia produced on the partially resistant varieties and inoculated on Merlot (INF, 0-1) measured on the grapevine varieties described in Table 5. Varieties were assigned to the following five groups: susceptible control 'Merlot' (test), varieties with no Rpv (none), or with Rpv3, Rpv10 or Rpv12. Values are means of data collected in a 3-year period and at three growth stage of grapevines, with 75 replicate leaf discs for each variety in each experiment. Values with different letters are significantly different according to the LSD test (P < 0.05). P-values indicate the significance of the effect of the variety in the ANOVA, and CV% are the coefficients of variation. Means followed by the same letter in the same column are not significantly different according to the Fisher-protected LSD test (P = 0.05).

Group	IFR		LP50		SPOR		IP		INF	
**test**	0.95	-	94.83	-	7257.0	-	3.27	-	0.77	-
**none**	0.90	-	98.84	-	4282.0	a	2.25	-	0.82	-
**Rpv3**	0.80	-	102.29	-	467.4	b	2.76	-	0.81	-
**Rpv10**	0.66	-	106.80	-	484.0	b	1.94	-	0.69	-
**Rpv12**	0.87	-	102.14	-	320.7	b	4.09	-	0.71	-
**P-value**	0.339		0.545		< 0.001		0.185		0.329	
**CV%**	13.05		4.41		121.44		29.88		7.61	

Figure S1: Example of calculation of the lesion size of downy mildew lesions on grapevine leaf discs. a) original image of a leaf disc photographed at 11 days post inoculation; b) delimitation of the perimeter of a single lesion; and c) demarcation of the lesion area that is measured (in mm^2) by the software Assess 2.0 (Image analysis software for plant disease quantification, by Lakhdar Lamari, APS PRESS, Saint Paul, Minnesota).

.

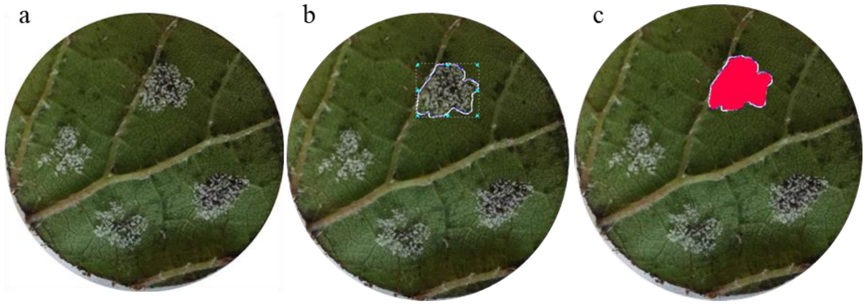

Supplement: Supplementary file 1 — Supplementary information. [file 41598_2020_57482_MOESM1_ESM.pdf]
